# Influence of Vegetation Coverage and Climate Environment on Soil Organic Carbon in the Qilian Mountains

**DOI:** 10.1038/s41598-019-53837-4

**Published:** 2019-11-26

**Authors:** Qiaozhuo Wan, Guofeng Zhu, Huiwen Guo, Yu Zhang, Hanxiong Pan, Leilei Yong, Huiying Ma

**Affiliations:** 1 College of Geography and Environment Science, Northwest Normal University, Lanzhou, 730070 Gansu China; 2 Gansu Engineering Research Center of Land Utilization and Comprehensio Consolidation, Lanzhou, 730070 China

**Keywords:** Element cycles, Hydrology, Hydrology, Hydrology, Hydrology

## Abstract

Studying the spatial distribution pattern of soil organic carbon and its influencing factors is essential for understanding the carbon cycle in terrestrial ecosystems. Soil samples from four active layers of typical vegetation types (Populus, subalpine shrubs, Picea crassifolia Kom, and alpine meadow) in the upper reaches of Shiyang River basin in the Qilian Mountains were collected to determine the soil organic carbon content and physicochemical properties. The results show the following: (1) There are significant differences in the vertical distribution of Soil organic carbon in the watershed, and the Soil organic carbon content decreases significantly with increasing soil depth. (2) Mainly affected by biomass, the organic carbon content of different vegetation types in different soil layers is as follows: Alpine meadow > Picea crassifolia Kom > Populus > Subalpine shrub, and the soil organic carbon content increases with increasing altitude. Under different vegetation types, the Soil organic content is the highest in the 0–30 cm soil profile, and the maximum value often appears in the 0–10 cm layer, then gradually decreases downward. (3) When soil organic carbon is determined in different vegetation types in the study area, the change of hydrothermal factors has little effect on soil organic carbon content in the short term.

## Introduction

The soil carbon pool is the largest carbon pool in terrestrial ecosystems. The accurate estimation of soil carbon pool reserves is of great significance to correctly evaluate the role of soil in the terrestrial ecosystem carbon cycle, global carbon cycle and global environmental change. The soil carbon pool storage reaches 1500–2500 Pg^1^ within global terrestrial ecosystems. Such an amount is twice the value of the atmospheric carbon pool^2^. Small changes in the soil organic carbon (SOC) pool can lead to significant changes in atmospheric carbon dioxide concentration^3^ and have an important impact on the global terrestrial ecosystem carbon cycle^4^. Soils in terrestrial ecosystems at high latitudes and high altitudes are in low-temperature environments and are sensitive to temperature changes. Warming may accelerate the decomposition of SOC in these regions and have a positive feedback effect on global climate change^5–7^. SOC content greatly affects the structure and properties of soils, thus affecting the productivity of vegetation. Studies have shown that SOC content mainly depends on surface vegetation and land use types^8^ and has a significant correlation with the residual leaves of plants entering the soil and with the microbial species in the soil^9^. Understanding the distribution of SOC in the soil surface and its influencing factors will help further improve the understanding of the mechanism of the underground carbon cycle and further enhance the understanding of the function of the soil carbon sink.

The spatial variability of SOC is quite complex. This variability is greatly influenced by environmental and human factors^10,11^, which directly or indirectly controls changes in the soil carbon pool. Therefore, the SOC spatial distribution depends on the relative contributions of different factors, such as vegetation, climate, soil, and parent material, and their interaction often leads to overlapping effects on its spatial distribution pattern^12^. Studies on the regional carbon cycle show that SOC accumulation and decomposition rates vary significantly under different hydrothermal conditions^13,14^. Many studies have emphasized the relationship between SOC and environmental factors^15^, but these studies rarely consider the SOC content in deeper soil^16^. Studies have shown that approximately 60% of SOC is located in 0–20 cm deep topsoil^17^ and is of great importance in the fragile ecosystems in semi-arid areas. However, the organic carbon in deep soil plays an important role in soil carbon storage because of its large reserve capacity; thus, it is necessary to understand the relationship between the vertical distribution of SOC and environmental factors^18^.

At present, the study of the spatial variability of soil properties has become one of the hotspots in the fields of soil science and ecological science^19^. The results show that SOC content is influenced by climate, vegetation, soil physical and chemical properties and human activities^20^. At present, research on SOC spatial variability and its influencing factors has mostly focused on different land use patterns and soil management measures in flat areas, while research on SOC spatial heterogeneity and its causes is rare in areas with complex topography and diverse vegetation types^21^. The study of the spatial heterogeneity of SOC is the basis of regional differentiation of soil hydrological processes and serves as an indirect parameter for input of distributed eco-hydrological models. Therefore, this information is of great significance for the study of spatial heterogeneity of SOC.

The Qilian Mountains are located in the northeastern margin of the Qinghai-Tibet Plateau, which consists of a series of parallel mountains and valleys from northwest to southeast. The average altitude of most mountains is over 4000 m, the vertical zonality of the vegetation is obvious; from low to high altitudes, and the vegetation is grassland, forest grassland, subalpine shrub meadow and sparse vegetation of alpine ice and snow. In the Qilian Mountains, studies on SOC have been carried out in some areas^22,23^; however, few authors have studied the vertical distribution pattern of SOC profiles and the quantitative relationship between SOC relative content at different levels and environmental factors and soil factors based on several representative profiles. This paper uses the Xiying River basin in the northern part of Lenglongling in the Qilian Mountains as the study area, discusses the variation characteristics of SOC in different soil depths, and analyzes the relationship with soil thermal and hydrological factors, aiming to analyze the influence of vegetation types and topography on the heterogeneity of the SOC spatial heterogeneity to provide basic information for the study of the regional environment.

## Overview of the Study Area

**Figure 1:**
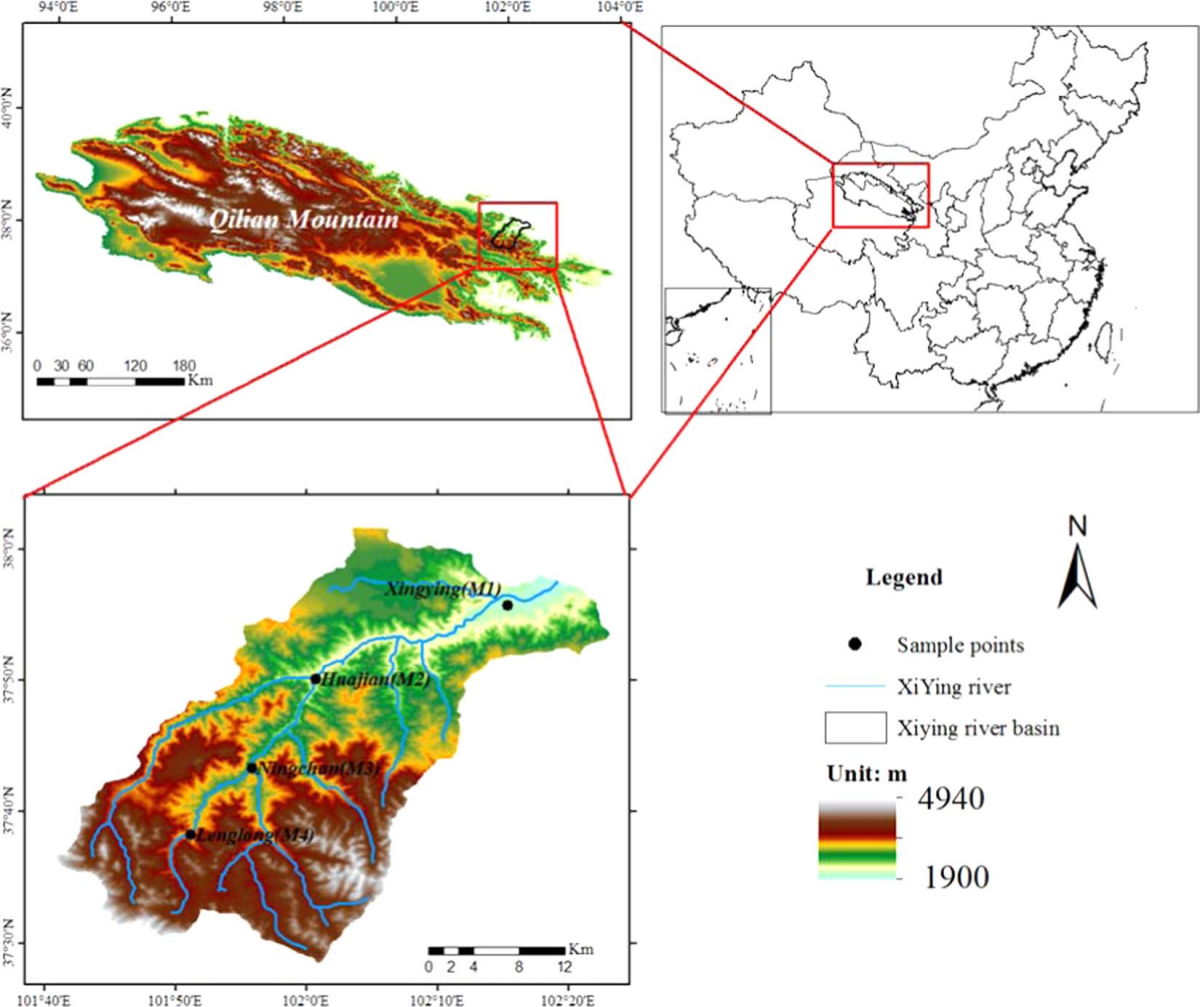
Overview of the Xiying River basin research area.

This paper uses the Xiying River basin in the Qilian Mountains as the research object (Fig. 1). The Xiying River is located in the upper reaches of the Shiyang River, ranging from 101°41′~102°04′E, 37°30′~37°52′N. The river originates from the northern slope of the Lenglongling Mountains in the Qilian Mountains. From southwest to northeast, the river collects the Shuiguan River, Ningchang River, Xiangshui River, and Tuta River and finally enters Xiying Reservoir. The flood season is mainly in spring and summer. The temperature increases in spring, the snow and ice melt in the mountainous areas, and the spring flood season occurs. The precipitation in summer is concentrated, forming a flood peak. Located in the alpine and semi-arid region of the Qilian Mountains, with an altitude of 1900–4900 m, the basin has a continental temperate arid climate, strong solar radiation, long sunshine duration, large temperature difference between day and night, less drought and rain in the basin, and precipitation is concentrated in summer, with an annual precipitation of 300–600 mm, strong evaporation and an annual evaporation of 700–1200 mm. The vertical zonality of vegetation in the basin is obvious, and the upper reaches are mainly forest grassland and subalpine shrub meadow vegetation belts. The middle and lower reaches are mainly bare land with poor vegetation coverage. The soil is mainly composed of lime soil, chestnut soil, alpine shrub meadow soil and desert soil.

## Data and Methods

### Sample investigation and sampling method

In the Xiying River basin, a tributary of the Shiyang River, Xiying (2097 m above sea level), Hujian Township (2323 m above sea level), Ningchan River (2721 m above sea level) and Lenglongling (3553 m above sea level) were selected as sampling points (Table 1) based on the elevation gradient and representative vegetation. The vegetation types are *Poplar*, *Picea crassifolia*, *Subalpine shrub* and *Alpine meadow*^24^. The sampling time is April - October 2018, on approximately the 20th of each month. The soil samples were collected by drilling with a 1-m-long drill. The soil samples were sampled once every 10-cm depth interval and put into sample bottles. The location of sampling points was accurately positioned by GPS, and the elevation and soil water content were recorded at the same time. Sample processing and determination were completed in the Engineering Research Center of Wetland Resources Protection and Industrial Development in Gansu Province.

**Table 1:** Sampling point locations in the Xiying River basin.

Sampling point	Sample size	Maximum Depth (cm)	Long (°E)	Lat (°N)	Alt (m)	T (°C)	P (mm)	Vegetation species
Xiying (M1)	47	90–100	102.26	37.93	2097	7.9	262.5	*Populus*
Huajian (M2)	73	90–100	102.01	37.83	2323	6.6	363.5	*Subalpine shrub*
Ningchan (M3)	50	70–80	101.93	37.72	2721	3.34	431.9	*Picea crassifolia*
Lenglong (M4)	46	80–90	101.85	37.64	3553	−0.19	595.1	*Alpine meadow*

### Soil data analysis

#### Soil organic carbon content

In the laboratory, soil samples were air dried, and pebbles and roots were removed using a 2-mm sieve. The SOC concentration was measured by potassium dichromate oxidation^25^. In the study, all soil profiles were divided into three levels: 0–30 cm, 30–50 cm, and 50–100 cm, and the SOC content was calculated accordingly.

$${C}_{2}=\frac{0.2\times 20}{{V}_{1}}$$

where C_2_ represents the ferrous sulfate standard solution concentration, and v1 represents the volume of consumption of ferrous sulfate.

$${\rm{C}}=\frac{({V}_{0}-V)\times {C}_{2}\times 0.03\times 1000}{M}$$

where C represents the organic carbon content g/kg, V0 represents the volume of ferrous sulfate consumed by two blank samples, V represents the volume of ferrous sulfate consumed by each sample, and M represents the quality of the sample.

#### Soil water content

The sampling time was April - October 2018, on approximately the 20th of each month. The soil samples were collected by drilling with a 1-m-long drill and were sampled once every 10-cm depth interval. The soil wet weight (M) is measured *in situ*. Then, the soil was brought back to the laboratory and dried at 105 °C for 24 h. The dry weight (Ms) of the soil was weighed, and the soil water content was calculated.

$${\rm{Soil}}\,{\rm{water}}\,content=\frac{M-{M}_{s}}{{M}_{S}}\times 100 \% $$

### Meteorological data

The meteorological data used were obtained from the four meteorological stations (Xiying, Huajian, Ningchan, Lenglong) in the Xiying River basin of Northwest Normal University. The main data obtained are the monthly average temperature and the monthly average precipitation.

### Data statistics

All data in this paper were analyzed by SPSS21 statistical software. One-way ANOVA was used to analyze the differences in soil organic carbon data variables among different vegetation types. Graphs were made using Sigmaplot12.5 software.

## Results

### Statistical characteristics of SOC

The descriptive statistical results of SOC in the study area are shown in Table 2. The results showed that the SOC content in the study area was generally low, and the SOC content generally showed a decreasing trend with increasing soil depth. From the profile distribution, the content of the 0–10 cm layer was the highest and decreased with increasing soil depth. The coefficient of variation, C_V_, determines the degree of variation of random variables, that is, the spatial difference of SOC. It is generally considered that C_V_ < 0.1 represents weak variability, 0.1 < C_V_ < 1.0 represents medium variability, and C_V_ > 1.0 represents strong variability^26^. Table 2 shows that, except for 80–90 cm, all the other soil layers are moderately variable. For the entire study area, the average soil organic carbon content was 6.37 g/kg, and the coefficient of variation was 0.56.

**Table 2:** Descriptive statistical parameter characteristics of SOC (g/kg).

Depth (cm)	Number of samples	Average value	Maximum	Minimum	Standard variance	Coefficient of variation	Skewness	Kurtosis
0–10	35	7.93	35.64	0.60	7.21**	0.91	2.31	6.09
10–20	34	6.79	15.55	0.30	3.91**	0.58	0.12	−0.87
20–30	33	6.77	22.37	1.20	4.92**	0.72	1.24	1.67
30–40	34	6.67	20.77	0.30	4.75**	0.71	1.27	1.31
40–50	26	5.91	16.70	0.59	4.51**	0.76	0.98	0.18
50–60	21	5.63	16.52	1.82	2.21**	0.39	0.27	−0.14
60–70	17	4.69	10.47	0.60	2.68**	0.57	0.62	0.14
70–80	11	3.57	7.48	0.60	2.66**	0.75	0.57	−1.40
80–90	7	3.43	6.26	2.72	3.55**	1.03	−1.45	1.92
90–100	4	2.56	6.96	0	1.52**	0.59	1.58	2.95

Note: **represents that the difference between sample points was significant (P < 0.05).

### Difference analysis of SOC in different vegetation cover areas

**Figure 2:**
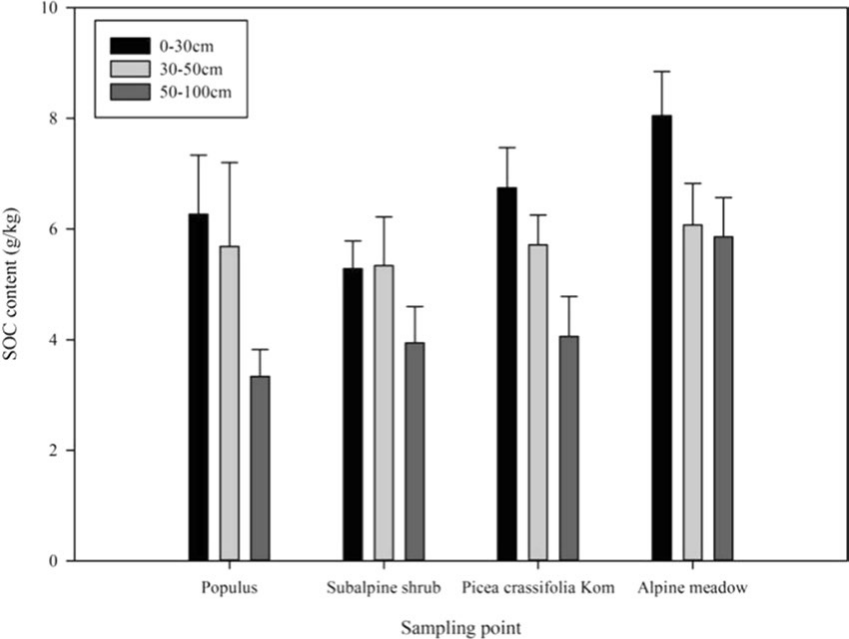
Difference analysis of SOC in different vegetation cover areas of the Xiying River basin.

Overall, the SOC decreased significantly with increasing soil depth in terms of the vertical distribution (Fig. 2). The average SOC content in the upper layer (0–30 cm) of the Xiying River basin was 7.17 g/kg, which was significantly lower than the average SOC content in the surface layer (18.4 g/kg) and the national average (18.6 g/kg)^27^. The average SOC content in the bottom layer (30–100 cm) was 5.59 g/kg, and the SOC content of the 0–30 cm layer accounted for 55.9% of the value of the top (1 m) soil layer. There were significant differences in SOC between the upper and lower levels (P < 0.01). The SOC content of the four vegetation types decreased with increasing soil depth (Fig. 2). Among them, the SOC content of Alpine meadow was the highest, with an average of 6.89 g/kg, followed by that of Qinghai spruce, with an average of 5.87 g/kg; the lowest value was in Subalpine shrubs, with an average of 4.98 g/kg. Figure 2 shows that the soil organic carbon content varies with different vegetation types at the same soil depth. From Fig. 2, it can be seen that, at the depth of 0–30 cm, the sequence of the organic carbon content is as follows: Alpine meadow > Picea crassifolia Kom > Populus > Subalpine shrub; among them, Alpine meadow has the most SOC, with a value of approximately 8.05 g/kg, and subalpine shrub has approximately 5.28 g/kg, which is the lowest value. In the 30–50 cm soil layer, the content of organic carbon is as follows: Alpine meadow > Picea crassifolia Kom > Populus > Subalpine shrub. The highest content is approximately 9.05 g/kg, and the lowest content is 5.24 g/kg. In the 50–100 cm soil layer, the content of organic carbon is as follows: Alpine meadow > Picea crassifolia Kom > Subalpine shrub > Populus, in which the content of organic carbon in Alpine meadow is approximately 5.85 g/kg and that in Populus is 3.33 g/kg. In general, the SOC (M4) of the Alpine meadow grassland was significantly higher than that of the other three plant types under the same soil depth (p < 0.01). And the SOC content of each soil layer in Qinghai spruce forest is close to that of poplar, but it is generally higher than poplar.

### Vertical distribution characteristics of SOC profiles in different vegetation types

**Figure 3:**
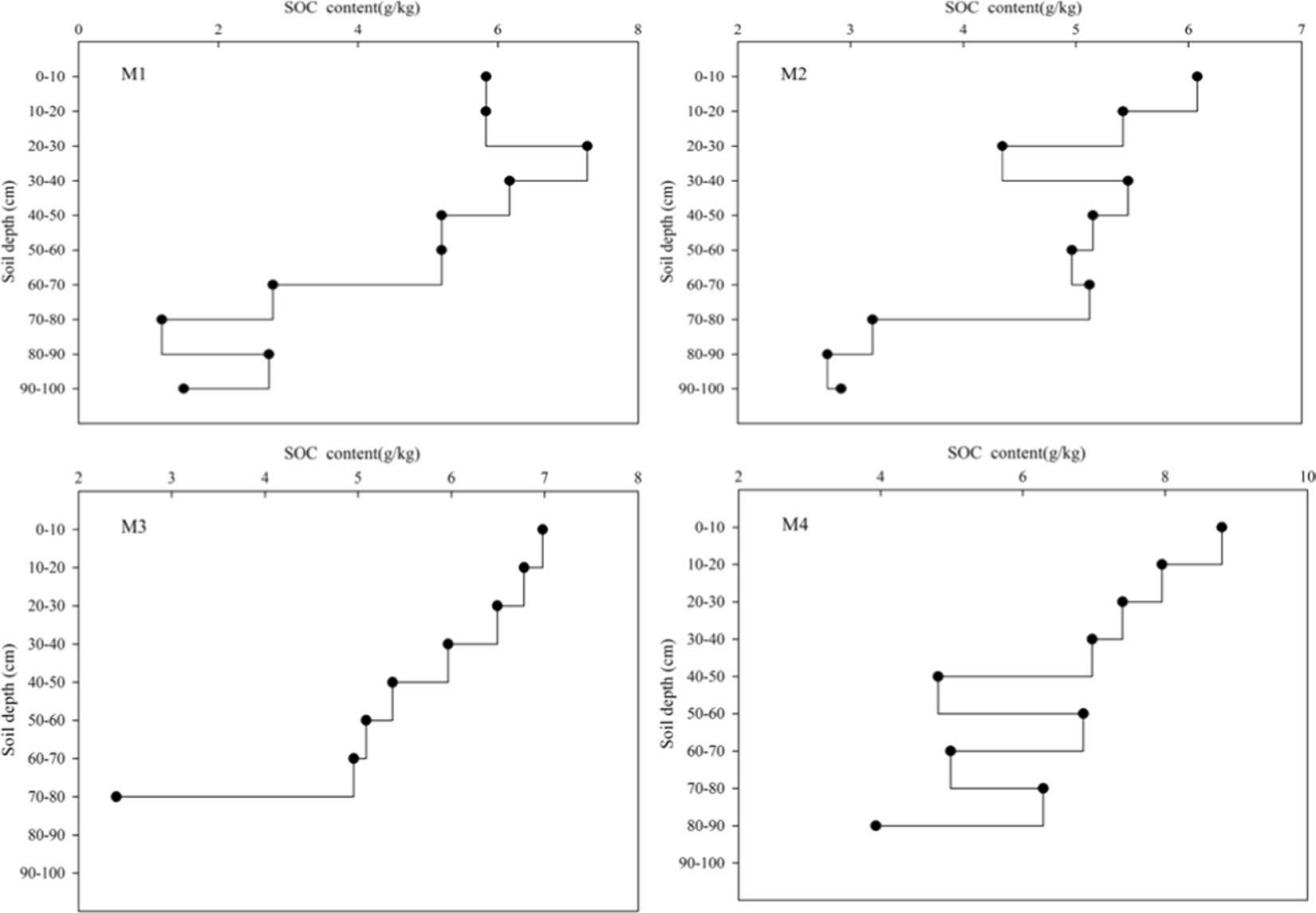
Vertical distribution of organic carbon profile in the Qilian Mountains.

The distribution of soil organic carbon in the soil profile is different due to the different root distribution, litter and artificial disturbance of soil under different vegetation types. As shown in Fig. 3, the SOC content in soils of different vegetation types showed a downward trend in the vertical direction. Poplars (M1) showed a significant change in SOC above 40 cm, reaching a maximum at 30 cm. This result is mainly because the organic carbon in the soil profile is directly affected by plant roots. Studies have shown that there are abundant vegetation roots in the 0–40 cm soil layer; thus, the SOC content in the upper layer of the soil is higher and decreases with increasing soil depth. The change in SOC content in shrubs (M2) was significant at 0–30 cm, and the maximum value appeared at 0–10 cm. Because shrub plants are low and roots are mostly distributed in the surface layer, there is more humus and higher organic carbon concentrations in the surface layer. The SOC content in Picea crassifolia Kom (M3) decreased significantly with increasing soil depth, and the maximum value appeared at 0–10 cm. This result is mainly because the soil surface is covered with litter and humus, and the decomposition of fallen matter is vigorous, releasing more nutrients. Over time, the litter and plant residues on the surface gradually decompose and transform into the deep soil layer with the leaching of rainwater and then gradually infiltrate into the deep soil layer. As a result, the deeper the soil organic carbon is, the lower the content of organic carbon is. The SOC content in Alpine meadow (M4) decreases with increasing soil depth. The SOC content in the 50–60 cm and 70–80 cm soil layers increased. This result is different from the common phenomenon in which the SOC content in the surface layer is higher than that in the lower layer. The main reason for this “abnormality” is that the number of soil samples investigated was too small. Moreover, the SOC content of Alpine meadow soil was generally higher than that of the other three grassland types. This result is because the surface soil vegetation of the meadow is more complicated, and the collected soil contains more fine plant roots and has more active microorganisms, which benefit the accumulation of organic carbon in the soil.

## Discussion

### Effects of different vegetation patterns on SOC

In Table 3, we summarize previous estimates of SOC reserves for different vegetation types at the regional scale. Table 3 shows that the average SOC content of the 0–100 cm depth in Xiying River basin of the Qilian Mountains is generally higher than that of the grassland types in Inner Mongolia but lower than those in Bayinbrook, Xinjiang, Qinghai-Tibet Plateau and Maqu grassland. As far as the Qilian Mountains are concerned, the SOC of each vegetation type in the upper reaches of the Heihe River is close to that of the Xiying River watershed.

**Table 3:** Comparison of SOC content and adjacent areas in the Qilian Mountains.

Research area	SOC (g/kg)				Reference
	Vegetation types	0–30 (cm)	30–50 (cm)	50–100 (cm)	
Inner Mongolia (106°-121°40′E, 40°20′-50°50′N)	Temperate grassland	4	5.19	6.68	^44^
	Desert steppe	2.3	3.1	4.01	
Bayinbulak, the middle section of the Tianshan Mountains, Xinjiang (83°431E, 42°541N)	Swamp meadow	137	—	—	^45^
	Alpine meadow	74	—	—	
	Meadow grassland	74.2	—	—	
Upper reaches of Heihe River in Qilian Mountains (98°-102° E, 38°-43°N)	Swamp meadow	—	—	49.5	^46^
	Alpine meadow	—	—	11.22	
	Alpine steppe	—	—	7.3	
Qinghai Tibet Plateau (74°-1042° E, 25°- 40° N)	Alpine meadow	68.09	—	—	^47,48^
	Alpine meadow type rangeland	58.02	—	—	
Grassland in Maqu (100°45′-102°29E, 33°06′-34°30′N)	Alpine grassland	45.55	—	—	^28^
Xiying River basin (101°41′~102°04′E, 37°30′~37°52′N)	Alpine meadow (M4)	8.05	6.07	5.85	This study
	Picea crassifolia Kom (M3)	6.73	5.71	4.06	
	Subalpine shrub (M2)	5.28	5.34	3.94	
	Populus (M1)	6.26	5.68	3.33	

Note: ‘–‘ indicates an absence of measurements.

From Table 3, it can be seen that in the 0–30 cm, 30–50 cm and 50–100 cm soil layers, the organic carbon content is as follows: Alpine meadow > Picea crassifolia Kom > Poplar > Subapine shrub. The SOC of the four vegetation types decreases with increasing soil depth, and the change in the organic carbon of Alpine meadow grassland is the largest, which indicates that the SOC of Alpine meadow is mainly distributed in the shallow surface layer, while that of Subalpine shrub is the smallest, which shows that the SOC in Subalpine shrubs is evenly distributed in different soil layers. The results of and showed that the SOC content of alpine shrubs in the middle part of the Qilian Mountains was the highest. This result may be related to the representativeness of the sampling points and the difference in the sampling area. Because the distribution of shrubs in the middle part of the Qilian Mountains has a long span in terms of elevation, the shrubs in the high elevation area form a strip distribution and form a mosaic distribution with arbor forests and grasslands in the low elevation area. In the shrub distribution area, temperature plays a controlling role in shrub growth. The shrub biomass in low altitude areas is higher than that in high altitude areas^23^. Therefore, if we choose a low altitude area, the shrub SOC content may be veryhigh^28^. If we sample in high altitude areas, the shrub SOC content may be low (in this paper); thus, the selection of sampling points has a great impact on the analysis of SOC of shrubs. In addition, precipitation and temperature were different during the sampling time, the soil microbial biomass and activity were enhanced by short-term sufficient moisture^29^ and suitable humidity^30^, and the sampling time also causes different SOC contents in the same vegetation type.

In this study, the SOC content of Alpine meadow soil was significantly higher than that of other vegetation types (Table 3), which was similar to existing research results^31,32^. Previous studies have shown that biomass differences among different vegetation types in the Qinghai-Tibet Plateau affect SOC density^33^. Field survey results showed that the aboveground biomass of alpine meadow was higher than that of other vegetation types^34^. Moreover, the growth and development of herbaceous plants consume soil nutrients, but the aboveground part of herbaceous plants died in the same year. After returning to the soil, soil SOC can be increased^35^.

Picea crassifolia forests in the Qilian Mountains are mainly distributed on shady and semi-shady slopes. The altitude of the upper line of forest distribution is 3200–3300 m. From the results of the study, the SOC content increases with increasing altitude, which is mainly due to the influence of precipitation and temperature changes. Because of the high precipitation, high soil moisture and low temperature in higher altitude areas, the decomposition and release of animal and plant residues are affected, and most of them are deposited in soil in the form of organic matter^36^. Different vegetation types have different effects on soil moisture^37^. The higher the tree is, the greater the canopy coverage is and the higher the water holding capacity is^38^. The canopy density of Poplar forests in the study area is high. The growth and development of herbaceous plants under forests are limited, thus reducing the consumption of soil moisture by plants.

### Relationship between SOC and climate and soil environmental factors

Under natural conditions, the SOC distribution is controlled by climate, vegetation, parent material and soil texture^31,39^. This study shows that changes in monthly mean precipitation and monthly mean temperature have little effect on soil organic carbon content. Vaughan^40^ and other scholars’ research resulted show that clay content, precipitation and temperature are related to soil organic carbon, but the content of lead oxide in the soil plays a decisive role. Fu Hua and Chen Yamin^41^ studied the soil organic carbon content of Alxa grassland in China. They showed that the main factors affecting the surface soil organic carbon were vegetation coverage and grassland productivity. And studies have shown that temperature and precipitation are secondary drivers of soil organic carbon dynamics at regional scale.Heng Tao^42^ found that the increase and decrease of monthly mean temperature did not change the soil organic carbon content in high-altitude mountainous areas.However, Domish^43^ observed that the increase of soil temperature would reduce the biomass carbon content of grassland soil. These differences may be caused by the differences of climate, vegetation and soil properties in different study areas. We need to point out that the impact of monthly average temperature on soil organic carbon content is complex and diverse. The change of monthly average temperature may lead to the increase or decrease of soil biomass.However, it is also possible that soil biomass will increase or decrease due to other environmental factors in the field, and the combined effects of various factors may offset the increase or decrease of soil biomass. Therefore, the change of soil organic carbon content is not significant.

Water is the limiting factor of plant production in alpine grassland, because the small increase of water can stimulate the biological productivity, thus contribute to the accumulation of SOC. The area selected in this study is the Alpine meadow distribution area in the Qilian Mountains, with high altitude, low temperature and high soil moisture. The increase or decrease of monthly average precipitation can increase or decrease soil moisture, but the range of soil moisture can meet the needs of biological activities, so precipitation has little effect on soil organic carbon.

In summary, the distribution of soil organic carbon content is the result of a combination of many influencing factors. And there is a strong interaction between the factors. If we want to understand the mechanism of soil organic carbon accumulation and its key influencing factors, we need to conduct more in-depth research on other factors affecting soil organic carbon such as topography of the study area, grazing, grassland reclamation and other human factors. Thereby, various influencing factors are organically combined to make a more accurate assessment of the reserves and directions of soil organic carbon in the ecosystem. In addition, soil organic matter requires a long-term process to change significantly. The study period is short, and the effects of temperature and precipitation changes on soil organic carbon and total nitrogen content are difficult to manifest.

### Relation between SOC and altitude

There are different vegetation types in the 2097–3553 m altitude zone in the study area, and the main vegetation types on the shady and sunny slopes are different. The influence of altitude on SOC content is mainly realized by the combination of water and heat. The temperature in the Qilian Mountains decreases with increasing altitude, with a decreasing rate of 0.58 °C/100 m, and precipitation increases with increasing altitude, with an increasing rate of 18.6 mm/100 m^36^. From the results of the study, the SOC content increased significantly with increasing altitude, mainly due to the influence of precipitation and temperature. As the elevation increases, precipitation increases and soil moisture increases; additionally, low temperature, which inhibits the activity of microorganisms, slows the decomposition rate of animal and plant residues and decreases the mineralization rate of SOC, causing organic carbon to be deposited in the soil and thus increasing the SOC reserves.

## Conclusions

Based on the analysis of the characteristics of SOC changes in the Xiying River watershed in the upper reaches of the Shiyang River in the Qilian Mountains on the northeastern margin of the Qinghai-Tibet Plateau, the following conclusions are drawn:

1. In terms of the vertical distribution, SOC decreased significantly with increasing soil depth. The average SOC content in the upper layer (0–30 cm) and the bottom layer (30–100 cm) of the Xiying River basin was 7.16 g/kg and 5.59 g/kg, respectively, and the SOC content in the 0–30 cm layer accounted for 55.9% of that of the total topsoil (1 m). There were significant differences in SOC between the upper and lower levels (P < 0.01).
2. The soil organic carbon content is as follows: Alpine meadow > Picea crassifolia Kom > Poplar > Subalpine shrub, under different vegetation patterns. The four vegetation types decreased with increasing soil depth, and the SOC in alpine meadow grassland changed the most, which indicated that SOC in alpine meadow was mainly distributed in the shallow surface layer, while SOC in subalpine shrubs changed the least, indicating that SOC in subalpine shrubs was evenly distributed in all soil layers.
3. When soil organic carbon is determined in different vegetation types in the study area, the change of hydrothermal factors has little effect on soil organic carbon content in the short term.
